# Supplementary material for: The impact of atypical intrahospital transfers on patient outcomes: a mixed methods study
Source: Sci Rep. 2023 Sep 18;13:15417. doi: 10.1038/s41598-023-41966-w (PMC10507077; doi:10.1038/s41598-023-41966-w)
Supplement: Supplementary file 1 — Supplementary Information. [file 41598_2023_41966_MOESM1_ESM.pdf]

Supplementary Information:

Univariable regression results:

Table 1a: Unadjusted average marginal effects for the relationship between atypical transfers, regular transfers, site transfers and increased length of stay derived from a gamma GLM with log link.

Variable	AME	Lower 95% CI	Upper 95% CI	p-value
Atypical transfers	3.34	3.06	3.68	<0.001
Regular transfers	3.09	2.99	3.20	<0.001
Site transfers	7.70	6.11	9.30	<0.001

Table 1b: Unadjusted odds ratios (OR) for the relationship between atypical, routine and site transfers and in-hospital mortality derived from logistic regressions.

Variable	OR	Lower 95% CI	Upper 95% CI	p-value
Atypical transfers	1.14	1.05	1.23	0.001
Regular transfers	1.18	1.16	1.20	0.001
Site transfers	1.00	0.91	1.09	0.98

Table 1c: Unadjusted odds ratios (OR) for the relationship between atypical, routine and site transfers and 30-day emergency readmission derived from logistic regressions.

Variable	OR	Lower 95% CI	Upper 95% CI	p-value
Atypical transfers	0.98	0.93	1.03	0.37
Regular transfers	1.02	1.00	1.03	0.02
Site transfers	1.02	0.96	1.08	0.51

Table 2: Full table of patient characteristics

	No atypical transfers (N=48064)	At least ones atypical transfer(N=7088)	Total (N=55152)
Routine ward transfers			
**Median**	1	0	1
**Q1, Q3**	1, 2	0, 2	1, 2.
Site transfers			
**Median**	0	0	0
**Q1, Q3**	0,0	0, 0	0, 0
Age (years)			
**18-39**	6273 (13.1%)	906 (12.8%)	7179 (13.0%)
**40-65**	15332 (31.9%)	2205 (31.1%)	17537 (31.8%)
**over 65**	26459 (55.0%)	3977 (56.1%)	30436 (55.2%)
Gender			
**Female**	22419 (46.6%)	3589 (50.6%)	26008 (47.2%)
**Male**	25645 (53.4%)	3499 (49.4%)	29144 (52.8%)
Length of stay (days)			
**Median**	7	9	7.2
**Q1, Q3**	3.3, 14.4	4.2, 19.6	3.5, 14.9
Elixhauser comorbidities (n, %)			
**>=5**	9219 (19.2%)	1338 (18.9%)	10557 (19.1%)
**0**	5254 (10.9%)	839 (11.8%)	6093 (11.0%)
**1-4**	33591(69.9%)	4911 (69.3%)	38502 (69.8%)
Discharge destination			
**Not usual place of residence**	7190 (15.0%)	1212 (17.1%)	8402 (15.2%)
**Usual place of residence**	40874 (85.0%)	5876 (82.9%)	46750 (84.8%)
Primary diagnosis count (n, %)			
**Mean (SD)**	1.2 (0.4)	1.3 (0.5)	1.2 (0.4)
**Range**	1.0 - 9.0	1.0 - 8.0	1.0 - 9.0
Intensive Care Admission			
**0**	44747 (93.1%)	6678 (94.2%)	51425 (93.2%)
**1**	3317 (6.9%)	410 (5.8%)	3727 (6.8%)
mortality			
**0**	45454 (94.6%)	6676 (94.2%)	52130 (94.5%)
**1**	2610 (5.4%)	412 (5.8%)	3022 (5.5%)
Weekend admission			
**FALSE**	36749 (76.5%)	5294 (74.7%)	42043 (76.2%)
**TRUE**	11315 (23.5%)	1794 (25.3%)	13109 (23.8%)
Out of hours admission (7pm-7am)			
**0**	25273 (52.6%)	3400 (48.0%)	28673 (52.0%)
**1**	22791 (47.4%)	3688 (52.0%)	26479 (48.0%)
Diagnostic imaging procedures (n, %)			
**0**	23701 (49.3%)	2646 (37.3%)	26347 (47.8%)
**0-3**	22206 (46.2%)	3891 (54.9%)	26097 (47.3%)
**4-6**	1835 (3.8%)	440 (6.2%)	2275 (4.1%)
**Over 6**	322 (0.7%)	111 (1.6%)	433 (0.8%)
Minor surgical procedures (n, %)			
**0**	40339 (83.9%)	5881 (83.0%)	46220 (83.8%)
**1-2**	7193 (15.0%)	1103 (15.6%)	8296 (15.0%)

**3-5**	473 (1.0%)	87 (1.2%)	560 (1.0%)
**Over 5**	59 (0.1%)	17 (0.2%)	76 (0.1%)
Major surgical procedures (n, %)			
**0**	41334 (86.0%)	5857 (82.6%)	47191.0 (85.6%)
**1-3**	6198 (12.9%)	1168 (16.5%)	7366.0 (13.4%)
**4-6**	453 (0.9%)	55 (0.8%)	508.0 (0.9%)
**Over 6**	79 (0.2%)	8 (0.1%)	87.0 (0.2%)
Ethnicity code description (n, %)			
**African**	1816 (3.8%)	243 (3.4%)	2059.0 (3.7%)
**Any other Asian background**	2021 (4.2%)	277 (3.9%)	2298.0 (4.2%)
**Any other Black background**	998 (2.1%)	159 (2.2%)	1157.0 (2.1%)
**Any other ethnic group**	6691 (13.9%)	1013 (14.3%)	7704.0 (14.0%)
**Any other mixed background**	289 (0.6%)	53 (0.7%)	342.0 (0.6%)
**Any other White background**	5629 (11.7%)	843 (11.9%)	6472.0 (11.7%)
**Bangladeshi**	267 (0.6%)	37 (0.5%)	304.0 (0.6%)
**British**	16371 (34.1%)	2414 (34.1%)	18785.0 (34.1%)
**Caribbean**	2366 (4.9%)	368 (5.2%)	2734.0 (5.0%)
**Chinese**	235 (0.5%)	28 (0.4%)	263.0 (0.5%)
**Indian**	2093 (4.4%)	262 (3.7%)	2355.0 (4.3%)
**Irish**	1841 (3.8%)	285 (4.0%)	2126.0 (3.9%)
**Pakistani**	474 (1.0%)	66 (0.9%)	540.0 (1.0%)
**Unknown**	6555 (13.6%)	980 (13.8%)	7535.0 (13.7%)
**White and Asian**	114 (0.2%)	15 (0.2%)	129.0 (0.2%)
**White and Black African**	82 (0.2%)	16 (0.2%)	98.0 (0.2%)
**White and Black Caribbean**	222 (0.5%)	29 (0.4%)	251.0 (0.5%)
Major diagnostic category (n, %)			
**Certain infectious and parasitic diseases**	3371 (7.0%)	420 (5.9%)	3791 (6.9%)
**Congenital malformations, deformations and chromosomal abnormalities**	26 (0.1%)	5 (0.1%)	31 (0.1%)
**Diseases of the blood and blood-forming organs and certain disorders involving the immune mechanism**	1023 (2.1%)	91 (1.3%)	1114 (2.0%)
**Diseases of the circulatory system**	8985 (18.7%)	1253 (17.7%)	10238 (18.6%)
**Diseases of the digestive system**	4223 (8.8%)	543 (7.7%)	4766 (8.6%)
**Diseases of the ear and mastoid process**	54 (0.1%)	19 (0.3%)	73 (0.1%)
**Diseases of the eye and adnexa**	147 (0.3%)	33 (0.5%)	180 (0.3%)
**Diseases of the genitourinary system**	4095 (8.5%)	575 (8.1%)	4670 (8.5%)
**Diseases of the musculoskeletal system and connective tissue**	1006 (2.1%)	281 (4.0%)	1287 (2.3%)
**Diseases of the nervous system**	1114 (2.3%)	297 (4.2%)	1411 (2.6%)
**Diseases of the respiratory system**	7717 (16.1%)	859 (12.1%)	8576 (15.5%)
**Diseases of the skin and subcutaneous tissue**	906 (1.9%)	136 (1.9%)	1042 (1.9%)
**Endocrine, nutritional and metabolic diseases**	1262 (2.6%)	166 (2.3%)	1428 (2.6%)

**External causes of morbidity**	5 (0.0%)	0 (0.0%)	5 (0.0%)
**Factors influencing health status and contact with health services**	129 (0.3%)	18 (0.3%)	147 (0.3%)
**Injury, poisoning and certain other consequences of external causes**	6018 (12.5%)	751 (10.6%)	6769 (12.3%)
**Mental, Behavioral and Neurodevelopmental disorders**	111 (0.2%)	22 (0.3%)	133 (0.2%)
**Neoplasms**	2722 (5.7%)	654 (9.2%)	3376 (6.1%)
**Symptoms, signs and abnormal clinical and laboratory findings, not elsewhere classified**	5150 (10.7%)	965 (13.6%)	6115 (11.1%)

Full multivariable regression results

Table 3: Multivariable generalised linear model (GLM) regression with a gamma distribution and log link predicting hospital length of stay (n = 55152). Results are presented as adjusted average marginal effects in days, with 95% CI based on standard errors clustered at the patient level (n = 38101).

variable	estimate	SE	lower 95% CI	upper 95% CI	p-value
(Intercept)	8.26	0.03	7.74	8.8	<0.001
Atypical transfers	1.22	0.01	1.19	1.24	<0.001
Regular transfers	1.14	0	1.13	1.15	<0.001
Site transfers	1.23	0.01	1.21	1.26	<0.001
Reference aged 18 to 40 years					
40-65	1.17	0.01	1.14	1.2	<0.001
Over 65	1.53	0.01	1.49	1.57	<0.001
Reference Over 5 Elixhauser comorbidities					
factor(index)0	0.68	0.02	0.66	0.71	<0.001
1-4	0.85	0.01	0.84	0.87	<0.001
Reference not weekend admission					
Weekend admission	1.01	0.01	0.99	1.03	0.16
Reference Discharge not to usual place of residence					
Usual place of residence	0.64	0.01	0.63	0.66	<0.001
Reference no ICU attendance					
ICU admission	1.17	0.02	1.13	1.22	<0.001
Reference female					
Male	0.97	0.01	0.95	0.99	<0.001
Reference 1 primary diagnosis					
2-3	1.43	0.01	1.4	1.47	<0.001
Over 3	2.29	0.08	1.96	2.66	<0.001
Reference 0 intermediate surgical procedures					
1-3	1.62	0.01	1.6	1.65	<0.001
4-6	2.85	0.02	2.73	2.98	<0.001
Over 6	3.69	0.05	3.35	4.05	<0.001
Reference 0 minor surgical procedures					
1-2	1.12	0.01	1.09	1.15	<0.001
3-5	1.35	0.04	1.24	1.46	<0.001
Over 5	1.37	0.11	1.1	1.7	<0.001
Reference 0 intermediate surgical procedures					
1-4	1.07	0.01	1.05	1.09	<0.001
5-7	1.53	0.05	1.4	1.67	<0.001
8-10	1.58	0.06	1.4	1.79	<0.001
Over 10	2.53	0.09	2.1	3.05	<0.001

Reference 0 major surgical procedures					
1-3	1.37	0.01	1.34	1.41	<0.001
4-6	1.77	0.04	1.62	1.93	<0.001
Over 6	2.17	0.1	1.77	2.66	<0.001
Reference admission between 7am and 7pm					
Out-of-hours admission	1.06	0.01	1.04	1.08	<0.001
Reference African ethnicity code					
Any other Asian background	0.85	0.03	0.8	0.9	<0.001
Any other Black background	0.94	0.03	0.88	1.01	0.09
Any other ethnic group	0.87	0.02	0.83	0.91	<0.001
Any other mixed background	0.82	0.06	0.73	0.91	<0.001
Any other White background	0.86	0.02	0.82	0.91	<0.001
Bangladeshi	0.82	0.06	0.73	0.91	<0.001
British	0.92	0.02	0.88	0.96	<0.001
Caribbean	0.98	0.03	0.93	1.04	0.58
Chinese	0.88	0.06	0.78	0.99	0.04
Indian	0.91	0.03	0.86	0.97	<0.001
Irish	0.94	0.03	0.88	0.99	0.03
Pakistani	0.85	0.05	0.77	0.93	<0.001
Unknown	0.87	0.02	0.83	0.92	<0.001
White and Asian	0.75	0.09	0.63	0.88	<0.001
White and Black African	0.81	0.1	0.67	0.98	0.03
White and Black Caribbean	0.95	0.06	0.84	1.08	0.43
Reference certain infectious and parasitic diseases ICD-10 chapter					
Congenital malformations, deformations and chromosomal abnormalities	0.83	0.17	0.6	1.17	0.29
Diseases of the blood and blood-forming organs and certain disorders involving the immune mechanism	1.07	0.03	1	1.14	0.05
Diseases of the circulatory system	0.71	0.02	0.68	0.73	<0.001
Diseases of the digestive system	0.68	0.02	0.65	0.71	<0.001
Diseases of the ear and mastoid process	0.61	0.11	0.49	0.76	<0.001
Diseases of the eye and adnexa	0.5	0.07	0.43	0.58	<0.001
Diseases of the genitourinary system	0.97	0.02	0.93	1.01	0.11
Diseases of the musculoskeletal system and connective tissue	0.95	0.03	0.89	1.01	0.08
Diseases of the nervous system	0.91	0.03	0.85	0.96	<0.001
Diseases of the respiratory system	0.92	0.02	0.89	0.96	<0.001

Diseases of the skin and subcutaneous tissue	1.05	0.03	0.98	1.12	0.16
Endocrine, nutritional and metabolic diseases	0.81	0.03	0.76	0.86	<0.001
factor(chapter)External causes of morbidity	0.94	0.42	0.41	2.17	0.89
Factors influencing health status and contact with health services	0.69	0.08	0.59	0.81	<0.001
Injury, poisoning and certain other consequences of external causes	0.91	0.02	0.87	0.95	<0.001
Mental, Behavioral and Neurodevelopmental disorders	1.02	0.08	0.86	1.2	0.85
Neoplasms	0.77	0.02	0.74	0.81	<0.001
Symptoms, signs and abnormal clinical and laboratory findings, not elsewhere classified	0.68	0.02	0.65	0.71	<0.001

Table 4: Multivariable logistic regression predicting in-hospital mortality (n = 55000). Results are presented as adjusted odds ratios, with 95% CI based on standard errors clustered at the patient level.

**variable**	**estimate**	**SE**	**lower 95% CI**	**upper 95% CI**	**p-value**
(Intercept)	0	0.24	0	0.01	<0.001
Atypical transfers	0.97	0.04	0.89	1.05	0.41
Regular transfers	0.99	0.01	0.96	1.01	0.37
Site transfers	0.72	0.06	0.64	0.80	<0.001
Reference aged 18 to 40 years					
40-65	3.26	0.16	2.38	4.46	<0.001
Over 65	8.61	0.16	6.33	11.71	<0.001
Reference 0 Elixhauser comorbidities					
Over 5	3.92	0.14	2.98	5.16	<0.001
1-4	1.87	0.14	1.43	2.45	<0.001
Reference not weekend admission					
Weekend admission	1.08	0.05	0.99	1.19	0.08
Reference female					
Male	0.98	0.04	0.9	1.06	0.55
Reference 1 primary diagnosis					
ICU admission	5.19	0.06	4.61	5.84	<0.001
Reference 1 primary diagnosis					
2-3	2.6	0.05	2.36	2.87	<0.001
Over 3	3.23	0.25	1.99	5.26	<0.001
Reference 0 imaging surgical procedures					

1-3	1.34	0.04	1.23	1.47	<0.001
4-6	1.87	0.09	1.58	2.22	<0.001
Over 6	2.66	0.16	1.96	3.61	<0.001
Reference 0 minor surgical procedures					
1-2	0.96	0.06	0.86	1.08	0.54
3-5	1.11	0.16	0.81	1.51	0.51
Over 5	2.09	0.32	1.11	3.95	0.02
Reference 0 intermediate surgical procedures					
1-4	1.09	0.05	0.99	1.2	0.08
5-7	1.34	0.19	0.92	1.96	0.13
8-10	1.1	0.3	0.61	2	0.75
Over 10	1.94	0.43	0.84	4.5	0.12
Reference 0 major surgical procedures					
1-3	0.71	0.07	0.62	0.81	<0.001
4-6	0.5	0.19	0.34	0.73	<0.001
Over 6	0.42	0.44	0.18	1	0.05
Reference admission between 7am and 7pm					
Out-of-hours admission	1.1	0.04	1.01	1.19	0.02
Reference African ethnicity code					
Any other Asian background	1.21	0.16	0.89	1.64	0.23
Any other Black background	0.82	0.21	0.54	1.24	0.35
Any other ethnic group	1.08	0.14	0.83	1.41	0.55
Any other mixed background	0.84	0.33	0.44	1.58	0.59
Any other White background	1.18	0.14	0.9	1.54	0.23
Bangladeshi	0.66	0.34	0.34	1.29	0.23
British	1.14	0.13	0.89	1.47	0.29
Caribbean	1.04	0.15	0.77	1.4	0.81
Chinese	1.18	0.29	0.66	2.08	0.58
Indian	1.04	0.16	0.77	1.41	0.81
Irish	1.07	0.15	0.79	1.44	0.68
Pakistani	1.07	0.25	0.66	1.74	0.77
Unknown	1.15	0.13	0.88	1.49	0.3
White and Asian	0.81	0.46	0.33	1.99	0.65
White and Black African	1.16	0.61	0.35	3.85	0.81
White and Black Caribbean	0.69	0.42	0.3	1.57	0.38
Reference certain infectious and parasitic diseases ICD-10 chapter					
Congenital malformations, deformations and chromosomal abnormalities	1.82	1.04	0.24	13.95	0.56
Diseases of the blood and blood-forming organs and certain disorders involving the immune mechanism	0.31	0.26	0.19	0.53	<0.001

Diseases of the circulatory system	0.63	0.07	0.54	0.73	<0.001
Diseases of the digestive system	0.55	0.1	0.45	0.66	<0.001
Diseases of the ear and mastoid process	0.19	1.02	0.03	1.38	0.1
Diseases of the eye and adnexa	0.16	0.77	0.03	0.7	0.02
Diseases of the genitourinary system	0.37	0.1	0.3	0.46	<0.001
Diseases of the musculoskeletal system and connective tissue	0.28	0.19	0.19	0.4	<0.001
Diseases of the nervous system	0.53	0.15	0.4	0.7	<0.001
Diseases of the respiratory system	0.94	0.07	0.82	1.09	0.43
Diseases of the skin and subcutaneous tissue	0.28	0.22	0.18	0.44	<0.001
Endocrine, nutritional and metabolic diseases	0.17	0.21	0.12	0.26	<0.001
Injury, poisoning and certain other consequences of external causes	0.36	0.1	0.29	0.43	<0.001
Mental, Behavioral and Neurodevelopmental disorders	0.45	0.47	0.18	1.14	0.09
Neoplasms	2.8	0.08	2.39	3.28	<0.001
Symptoms, signs and abnormal clinical and laboratory findings, not elsewhere classified	0.1	0.14	0.07	0.13	<0.001

Table 5: Multivariable logistic regression predicting emergency readmission (n = 52125). Results are presented as adjusted odds ratios, with 95% CI based on standard errors clustered at the patient level.

**variable**	**estimate**	**SE**	**lower 95% CI**	**upper 95% CI**	**p-value**
(Intercept)	0.06	0.12	0.05	0.08	<0.001
Atypical transfers	1.00	0.03	0.95	1.05	0.92
Regular transfers	1.02	0.01	1.00	1.04	0.02
Site transfers	1.03	0.03	0.97	1.09	0.38
Reference aged 18 to 40 years					
40-65	1.19	0.04	1.09	1.3	<0.001
Over 65	1.17	0.04	1.07	1.28	<0.001
Reference 0 Elixhauser comorbidities					
Over 5	2.79	0.06	2.5	3.11	<0.001
1-4	1.78	0.05	1.61	1.96	<0.001
Reference not weekend admission					
Weekend admission	1.01	0.03	0.96	1.07	0.63

Reference Discharge not to usual place of residence					
Usual place of residence	2.15	0.05	1.95	2.37	<0.001
Reference no ICU attendance					
ICU admission	0.74	0.06	0.66	0.83	<0.001
Reference female					
Male	1.08	0.02	1.03	1.13	<0.001
Reference 1 primary diagnosis					
2-3	1.08	0.04	1.00	1.15	0.04
Over 3	0.92	0.24	0.58	1.46	0.73
Reference 0 imaging surgical procedures					
1-3	0.8	0.03	0.76	0.85	<0.001
4-6	0.72	0.07	0.62	0.83	<0.001
Over 6	0.72	0.18	0.51	1.02	0.06
Reference 0 minor surgical procedures					
1-2	0.91	0.04	0.85	0.97	0.01
3-5	0.82	0.13	0.64	1.05	0.12
Over 5	0.76	0.36	0.37	1.54	0.44
Reference 0 intermediate surgical procedures					
1-4	0.92	0.03	0.87	0.98	0.01
5-7	0.97	0.15	0.72	1.3	0.84
8-10	1.1	0.2	0.75	1.63	0.62
Over 10	1.86	0.27	1.1	3.13	0.02
Reference 0 major surgical procedures					
1-3	0.77	0.04	0.71	0.83	<0.001
4-6	0.74	0.16	0.54	1.02	0.07
Over 6	0.89	0.37	0.43	1.86	0.76
Reference admission between 7am and 7pm					
Out-of-hours admission	0.94	0.02	0.9	0.99	0.01
Reference African ethnicity code					
Any other Asian background	0.93	0.08	0.79	1.09	0.36
Any other Black background	0.74	0.1	0.6	0.9	<0.001
Any other ethnic group	0.83	0.07	0.73	0.94	<0.001
Any other mixed background	1.03	0.16	0.76	1.39	0.87
Any other White background	0.86	0.07	0.75	0.98	0.02
Bangladeshi	0.91	0.16	0.66	1.25	0.55
British	1.04	0.06	0.92	1.17	0.56
Caribbean	1.02	0.07	0.88	1.18	0.79
Chinese	0.77	0.19	0.53	1.12	0.18
Indian	0.89	0.08	0.76	1.04	0.14
Irish	1.07	0.08	0.92	1.26	0.37
Pakistani	0.98	0.13	0.76	1.26	0.89
Unknown	0.71	0.07	0.63	0.82	0
White and Asian	0.53	0.29	0.3	0.93	0.03
White and Black African	1.06	0.27	0.63	1.79	0.82
White and Black Caribbean	1.56	0.16	1.15	2.12	<0.001
Reference certain infectious and parasitic diseases ICD-10 chapter					
Congenital malformations, deformations and chromosomal abnormalities	0.45	0.74	0.11	1.93	0.28

Diseases of the blood and blood-forming organs and certain disorders involving the immune mechanism	2.33	0.08	1.98	2.72	<0.001
Diseases of the circulatory system	0.67	0.05	0.6	0.74	0
Diseases of the digestive system	1.14	0.06	1.01	1.28	0.03
Diseases of the ear and mastoid process	0.78	0.35	0.4	1.55	0.48
Diseases of the eye and adnexa	0.61	0.27	0.36	1.04	0.07
Diseases of the genitourinary system	1.08	0.06	0.96	1.21	0.2
Diseases of the musculoskeletal system and connective tissue	0.95	0.09	0.8	1.13	0.58
Diseases of the nervous system	0.68	0.1	0.57	0.83	<0.001
Diseases of the respiratory system	0.98	0.05	0.89	1.09	0.74
Diseases of the skin and subcutaneous tissue	0.92	0.09	0.77	1.1	0.37
Endocrine, nutritional and metabolic diseases	0.99	0.08	0.84	1.15	0.86
Factors influencing health status and contact with health services	0.81	0.24	0.5	1.3	0.38
Injury, poisoning and certain other consequences of external causes	0.73	0.06	0.65	0.82	<0.001
Mental, Behavioral and Neurodevelopmental disorders	0.62	0.27	0.37	1.05	0.07
Neoplasms	1.65	0.06	1.46	1.87	<0.001
Symptoms, signs and abnormal clinical and laboratory findings, not elsewhere classified	0.97	0.06	0.87	1.08	0.58
Reference LOS 0-1 days					
1-2	0.81	0.07	0.7	0.93	<0.001
2-4	0.87	0.07	0.76	0.99	0.04
4-6	0.91	0.07	0.8	1.05	0.2
6-8	1.02	0.07	0.89	1.17	0.79
8-10	1.15	0.07	0.99	1.33	0.06
Over 10	1.31	0.07	1.15	1.49	<0.001

Sensitivity analyses:

Length of stay

Table 6a: Adjusted average marginal effects for the relationship between atypical transfers, regular transfers, site transfers and increased length of stay derived by a gamma GLM with log link, from observations whose standardised residuals were below 3 (n = 55003)

Variable	AME	SE	Lower 95%CI	Upper 95%CI	p-value
Atypical transfers	2.69	0.13	2.44	2.44	<0.001
Regular transfers	1.93	0.05	1.83	2.03	<0.001
Site transfers	3.01	0.15	2.73	3.30	<0.001

Table 6b: Adjusted average marginal effects for the relationship between atypical transfers, regular transfers, site transfers and increased length of stay derived by a gamma GLM with log link, from spells in which the patient was discharged alive and did not self-discharge (n=51210)

Variable	AME	SE	Lower 95%CI	Upper 95%CI	p-value
Atypical transfers	2.80	0.15	2.51	3.09	<0.001
Regular transfers	1.88	0.06	1.77	1.99	<0.001
Site transfers	2.93	0.17	2.60	3.26	<0.001

Table 6c: Adjusted average marginal effects for the relationship between atypical transfers, regular transfers, site transfers and increased length of stay derived by a gamma GLM with log link, with BUPA surgical categorisations used for procedure adjustment (n=55152).

Variable	AME	SE	Lower 95%CI	Upper 95%CI	p-value
Atypical transfers	3.03	0.15	2.75	3.32	<0.001
Regular transfers	2.13	0.05	2.03	2.24	<0.001
Site transfers	3.25	0.17	2.91	3.59	<0.001

Table 6d: Adjusted average marginal effects for the relationship between atypical transfers, regular transfers, site transfers and increased length of stay derived by a gamma GLM with log link, from spells in which the patient was free of infection or colonisation for the duration of their spell (n=47708)

Variable	AME	SE	Lower 95%CI	Upper 95%CI	p-value
Atypical transfers	2.91	0.16	2.60	3.21	<0.001
Regular transfers	1.95	0.06	1.83	2.07	<0.001
Site transfers	3.07	0.18	2.72	3.42	<0.001

Table 6e: Adjusted average marginal effects for the relationship between atypical transfers, regular transfers, site transfers and increased length of stay derived by a gamma GLM with log link, with approximate month of admission included in the adjustment set (n=55152)

Variable	AME	SE	Lower 95%CI	Upper 95%CI	p-value
Atypical transfers	2.81	0.14	2.52	3.10	<0.001
Regular transfers	1.93	0.05	1.82	2.04	<0.001
Site transfers	3.02	0.17	2.70	3.35	<0.001

Table 6f: Adjusted average marginal effects for the relationship between atypical transfers, regular transfers, site transfers and increased length of stay derived by a gamma GLM with log link, with alternative ward speciality categorisations obtained by using the *first* TFC associated with spells (n=55152)

Variable	AME	SE	Lower 95%CI	Upper 95%CI	p-value
Atypical transfers	2.58	0.15	2.28	2.87	<0.001
Regular transfers	1.97	0.05	1.86	2.07	<0.001
Site transfers	3.03	0.17	2.7	3.35	<0.001

Mortality

Table 7a: Adjusted odds ratios for the relationship between atypical transfers, regular transfers, site transfers and mortality derived by a logistic regression, from observations whose standardised residuals were below 3 (n = 54981)

Variable	Variable	SE	Lower 95%CI	Upper 95%CI	p-value
Atypical transfers	0.96	0.04	0.89	1.04	0.38
Regular transfers	0.98	0.01	0.96	1.01	0.23
Site transfers	0.71	0.06	0.63	0.80	<0.001

Table 7b: Adjusted odds ratios for the relationship between atypical transfers, regular transfers, site transfers and mortality derived by a logistic regression, with BUPA surgical categorisations used for procedure adjustment (n=55152).

Bupa surgical code categorisation:

Variable	Variable	SE	Lower 95%CI	Upper 95%CI	p-value
Atypical transfers	0.98	0.04	0.91	1.06	0.67
Regular transfers	0.99	0.01	0.97	1.02	0.61
Site transfers	0.73	0.06	0.65	0.82	<0.001

Table 7c: Adjusted odds ratios for the relationship between atypical transfers, regular transfers, site transfers and mortality derived by a logistic regression, from spells in which the patient was free of infection or colonisation for the duration of their spell (n=47584)

Variable	Variable	SE	Lower 95%CI	Upper 95%CI	p-value
Atypical transfers	0.96	0.04	0.88	1.04	0.29
Regular transfers	0.99	0.01	0.96	1.02	0.47
Site transfers	0.74	0.06	0.66	0.84	<0.001

Table 7d: Adjusted odds ratios for the relationship between atypical transfers, regular transfers, site transfers and mortality derived by a logistic regression, with approximate month of admission included in the adjustment set (n=55152)

Variable	Variable	SE	Lower 95%CI	Upper 95%CI	p-value
Atypical transfers	0.96	0.04	0.88	1.04	0.29
Regular transfers	0.99	0.01	0.96	1.02	0.41
Site transfers	0.71	0.06	0.63	0.80	<0.001

Table 7e: Adjusted odds ratios for the relationship between atypical transfers, regular transfers, site transfers and mortality derived by a logistic regression, with alternative ward speciality categorisations obtained by using the *first* TFC associated with spells (n= 55000)

Variable	Variable	SE	Lower 95%CI	Upper 95%CI	p-value
Atypical transfers	0.90	0.05	0.81	0.99	0.03
Regular transfers	0.99	0.01	0.97	1.02	0.52
Site transfers	0.72	0.06	0.64	0.80	<0.001

Readmission

The 30-day emergency readmission model did not obtain any observations whose standardised residuals were below 3, therefore this sensitivity analysis was omitted.

Table 8a: Adjusted odds ratios for the relationship between atypical transfers, regular transfers, site transfers and 30-day emergency readmission, with BUPA surgical categorisations used for procedure adjustment (n=52125).

Variable	Estimate	SE	Lower 95%CI	Upper 95%CI	p-value
Atypical transfers	1.00	0.03	0.95	1.06	0.89
Regular transfers	1.01	0.01	0.99	1.03	0.22
Site transfers	1.02	0.03	0.96	1.08	0.46

Table 8b: Adjusted odds ratios for the relationship between atypical transfers, regular transfers, site transfers and 30-day emergency readmission, from spells in which the patient was free of infection or colonisation for the duration of their spell (n=44988)

Variable	Estimate	SE	Lower 95%CI	Upper 95%CI	p-value
Atypical transfers	1.00	0.03	0.94	1.06	0.99
Regular transfers	1.03	0.01	1.01	1.05	0.01
Site transfers	1.00	0.04	0.93	1.07	0.96

Table 8c: Adjusted odds ratios for the relationship between atypical transfers, regular transfers, site transfers and 30-day emergency readmission, with approximate month of admission included in the adjustment set (n=52125)

Variable	Estimate	SE	Lower 95%CI	Upper 95%CI	p-value
Atypical transfers	1.00	0.03	0.95	1.05	0.90
Regular transfers	1.02	0.01	1.00	1.04	0.02
Site transfers	1.03	0.03	0.97	1.09	0.37

Table 8d: Adjusted odds ratios for the relationship between atypical transfers, regular transfers, site transfers and 30-day emergency readmission, with alternative ward speciality categorisations obtained by using the *first* TFC associated with spells (n= 52125).

Variable	Estimate	SE	Lower 95%CI	Upper 95%CI	p-value
Atypical transfers	1.00	0.03	0.95	1.05	0.92
Regular transfers	1.02	0.01	1.00	1.04	0.02
Site transfers	1.03	0.03	0.97	1.09	0.38

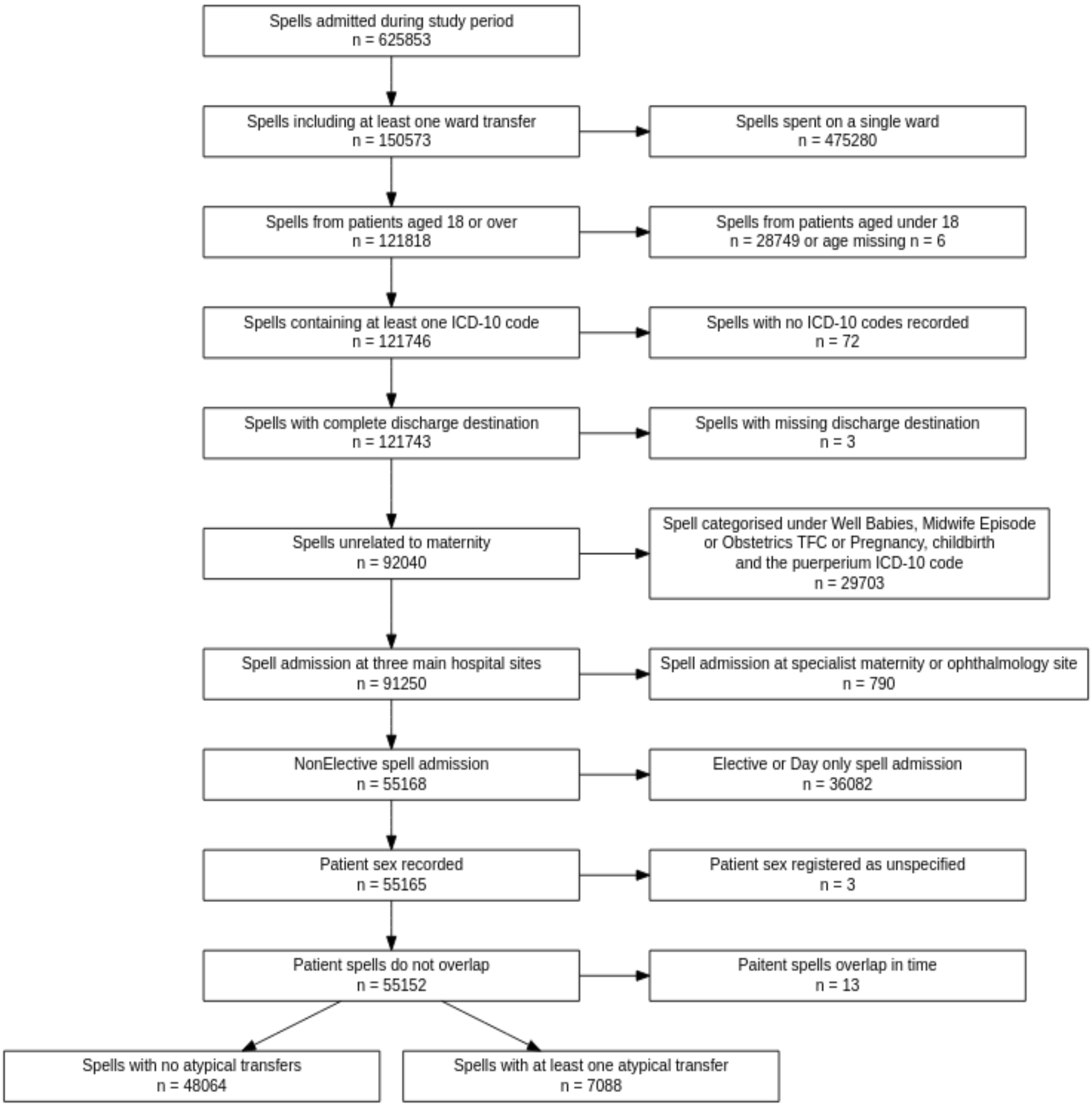

Figure 1: A flow diagram summarising the inclusion and exclusion criteria for the study participants. The final sample included 55,152 patient spells pertaining to 38,101 individual patients. Of these 7,088 (12.9%) spells included at least one atypical transfer.

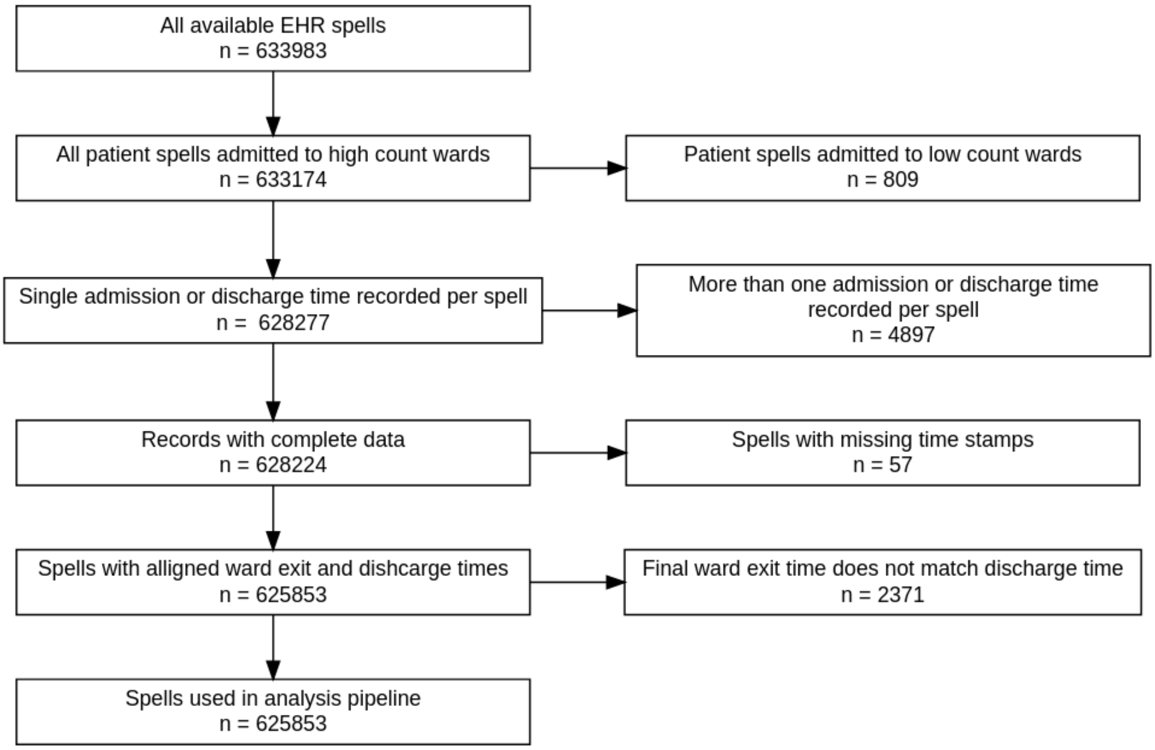

Figure 2: A flow diagram showing data cleaning decisions and the number of spells they affected prior to analysis.

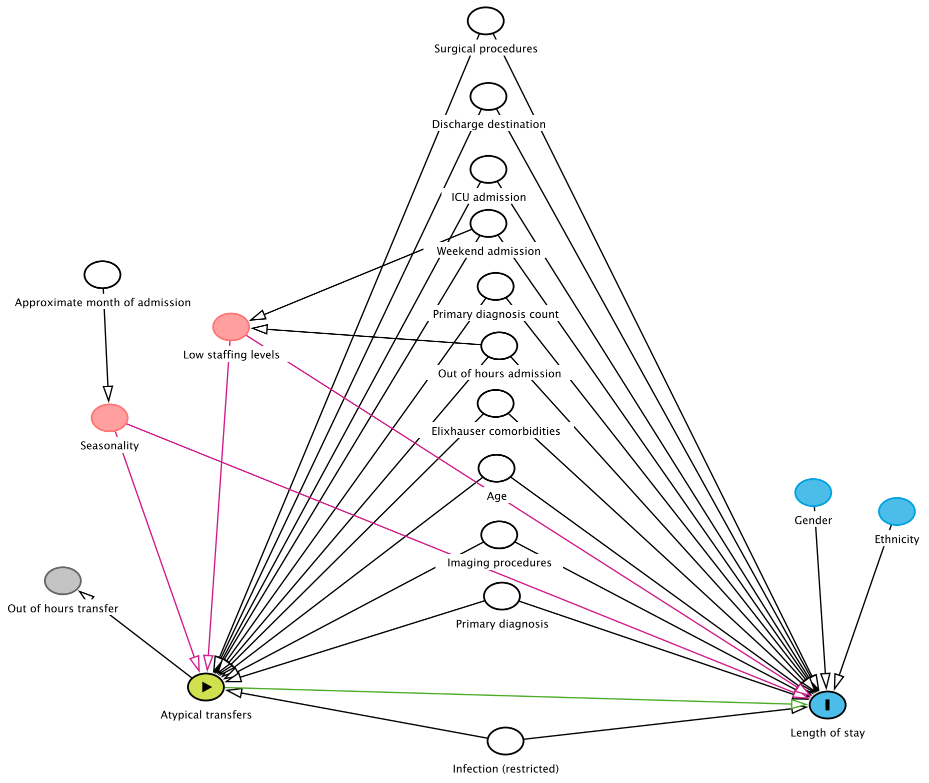

Figure 3: Example of a Directed Acyclic Graph (DAG) exploring the relationships between atypical transfers and length of stay.

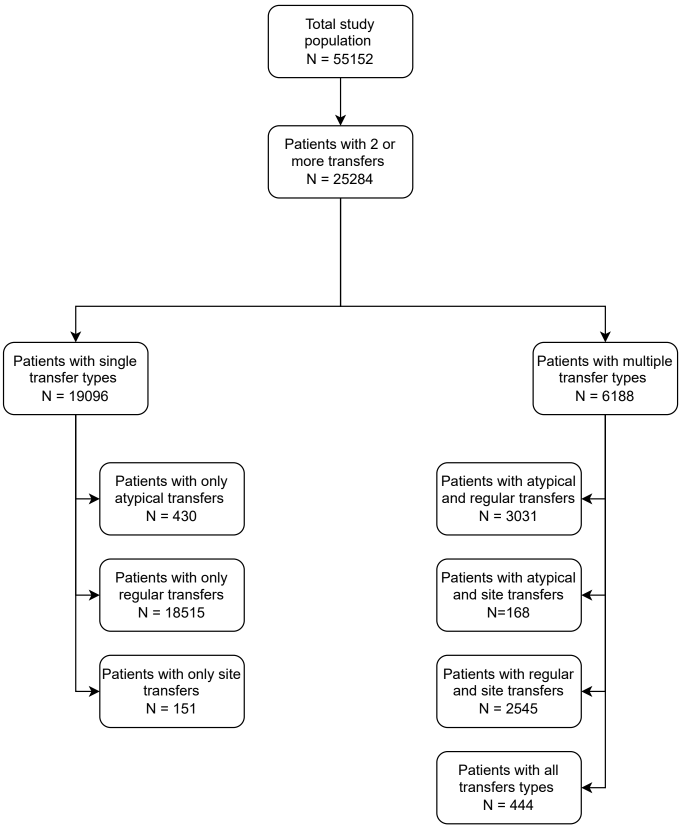

Figure 4: A breakdown of transfer type for patients with multiple transfers.

Note 1a: Identifying pregnant women

Women attending the hospital for reasons related to pregnancy were removed from the analysis if incurring the final TFC code *well babies*, *midwife episode* or *obstetrics* or the first chapter code *Pregnancy, childbirth and the puerperium.*

Note 1b: Categorisation of surgical procedures
Abbott et al., (2011) defined code lists of 'inclusive' procedures that could be considered surgery, such as minor surgery, interventional radiology procedures and diagnostic endoscopies.[2] The second 'intermediate' category included more severe procedures routinely undertaken in an operating theatre and/or under general or regional anaesthesia, while third 'restrictive' category included major procedures, defined as a complex procedure which may result in in tissue injury. While the authors originally defined categories not mutually exclusive (e.g. a restrictive procedure would also be counted in the inclusive category), in our analysis the codes were separated such that inclusive, intermediate and restrictive categories were distinct.

Note 1c: Alternative categorisation of surgical procedures by Bupa Schedule of Procedures

The Clinical Coding and Schedule Development Group (CCSD) schedule was developed to allow clinicians in private practice to be reimbursed for their work in the private healthcare sector. Surgical codes are allocated a complexity level (minor, intermediate, major, major plus and complex major) with an associated cost. While OPCS and CCSD codes cover the same major areas (or chapters), mapping OPCS and CCSD codes is challenging and cannot be achieved through a direct match. OPCS has more than 6,000 codes while CCSD has around 2,070, and therefore many OPCS codes may be associated with one CCSD code.

The Bupa Schedule of Procedures updated on 15 Oct 2021 by BUPA healthcare was used as a source of CCSD categories.[1] The 'hospital category' classification was selected to allocate procedures a severity level. The codes were matched to OPCS-4 codes through an exact match on the first 2 figures. A fuzzy match allowing a distance of 1 was used to match the remaining code figures to OPCS 4 codes. This resulted in a match for 2056 codes of the 5941 unique OPCS seen in the dataset.

References:

1. Bupa - Code Search main [Internet]. [cited 2022 Sep 20]. Available from: https://codes.bupa.co.uk/home

2. Abbott TEF, Fowler AJ, Dobbs TD, Harrison EM, Gillies MA, Pearse RM. Frequency of surgical treatment and related hospital procedures in the UK: a national ecological study using hospital episode statistics. BJA: British Journal of Anaesthesia [Internet]. 2017 Aug 1 [cited 2022 May 14];119(2):249–57. Available from: https://doi.org/10.1093/bja/aex137
